# Early-onset impairment of the ubiquitin-proteasome system in dopaminergic neurons caused by α-synuclein

**DOI:** 10.1186/s40478-020-0894-0

**Published:** 2020-02-14

**Authors:** Chris McKinnon, Mitchell L. De Snoo, Elise Gondard, Clemens Neudorfer, Hien Chau, Sophie G. Ngana, Darren M. O’Hara, Jonathan M. Brotchie, James B. Koprich, Andres M. Lozano, Lorraine V. Kalia, Suneil K. Kalia

**Affiliations:** 1 Krembil Research Institute, Toronto Western Hospital, University Health Network, 60 Leonard Avenue, Toronto, ON M5T 0S8 Canada; 2 Division of Neurosurgery, Department of Surgery, Toronto Western Hospital, University Health Network, University of Toronto, Toronto, ON Canada; 3 Division of Neurology, Department of Medicine, Toronto Western Hospital, University Health Network, University of Toronto, Toronto, ON Canada

**Keywords:** Parkinson’s disease, Ubiquitin-proteasome system, α-Synuclein, Neurodegeneration

## Abstract

Parkinson’s disease is a progressive neurodegenerative disorder characterised by the accumulation of misfolded α-synuclein in selected brain regions, including the substantia nigra pars compacta (SNpc), where marked loss of dopaminergic neurons is also observed. Yet, the relationship between misfolded α-synuclein and neurotoxicity currently remains unclear. As the principal route for degradation of misfolded proteins in mammalian cells, the ubiquitin-proteasome system (UPS) is critical for maintenance of cellular proteostasis. Misfolded α-synuclein impairs UPS function and contributes to neuronal death in vitro. Here, we examine its effects in vivo using adeno-associated viruses to co-express A53T α-synuclein and the ubiquitinated reporter protein Ub^G76V^-GFP in rat SNpc. We found that α-synuclein over-expression leads to early-onset catalytic impairment of the 26S proteasome with associated UPS dysfunction, preceding the onset of behavioural deficits and dopaminergic neurodegeneration. UPS failure in dopaminergic neurons was also associated with selective accumulation of α-synuclein phosphorylated at the serine 129 residue, which has previously been linked to increased neurotoxicity. Our study highlights a role for α-synuclein in disturbing proteostasis which may contribute to neurodegeneration in vivo*.*

## Introduction

Parkinson’s disease (PD) is a progressive neurodegenerative disease characterized by death of dopaminergic neurons in the substantia nigra pars compacta (SNpc) [21]. Impaired proteostasis is thought to play a central role in PD etiology due to intraneuronal accumulation of abnormal protein aggregates, composed primarily of misfolded α-synuclein, termed Lewy bodies and neurites [20]. These neuropathological hallmarks are features of the common idiopathic form of PD and rare inherited forms, such as autosomal dominant familial PD due to missense mutations (e.g., A53T) or multiplications of the *SNCA* gene encoding α-synuclein [38]. While Lewy bodies were originally postulated to be the neurotoxic aggregates of α-synuclein, cases of PD lacking Lewy pathology as well as identification of different α-synuclein aggregation products have implicated α-synuclein oligomers or small fibrils as the more likely neurotoxic aggregates [20]. Yet, the pathological link between α-synuclein oligomers and dopaminergic neuron dysfunction and death remains elusive. It is increasingly recognised that disruption of cellular proteostasis is a common feature across laboratory models of PD, with evidence of disruption in molecular chaperone proteins, the autophagy-lysosome pathway (ALP) and the ubiquitin-proteasome system (UPS) at late stages of disease [19]. The relative contribution of each of these cellular protein quality control pathways to early stages of disease pathogenesis remains uncertain. In the present study, we investigated whether expression of mutant α-synuclein is associated with early dysfunction of the UPS, which could contribute to the progressive proteostasis failure observed in PD.

The UPS is the major pathway for proteolytic degradation in mammalian cells. In this system, proteins are tagged for degradation by the covalent conjugation of polyubiquitin chains. These chains are recognized by the 19S regulatory particle of the proteasome which directs the substrate into the 20S core particle for degradation into short peptides [30]. Comparison of brain tissue from PD patients with samples from healthy controls has revealed reduced rates of proteasome catalytic activity and lower levels of certain proteasome subunits [1, 31–33]. This difference could reflect a direct effect of misfolded α-synuclein on UPS function or reflect a global failure of cellular proteostasis in advanced stages of disease. The former is supported by evidence from in vitro studies where overexpression of wild-type [41] or mutant α-synuclein [42, 43] inhibited proteasome activity in lysates from cultured cells. More recent studies have employed fluorescent reporter substrates to measure UPS activity in the more physiological context of intact cells. In dopaminergic SH-SY5Y cells, overexpression of α-synuclein is associated with elevated levels of the GFP-CL1 [36] and Ub^G76V^-GFP UPS reporter substrates. The degree of UPS dysfunction observed appears to be more pronounced with mutant (e.g. A53T) compared with wild-type α-synuclein [36]. In addition, comparison of the effect of α-synuclein expression on UPS activity in different cultured cell lines suggests that vulnerability to UPS dysfunction may be cell-type specific. As a major degradation pathway for clearance of α-synuclein in vivo [14]*,* dysfunction of the UPS could precipitate rising levels of α-synuclein in affected neurons. Consistent with this hypothesis, UPS inhibition in vivo is sufficient to replicate key hallmarks of PD neuropathology. For example, pharmacological inhibition of the proteasome has been found to induce dopaminergic neurodegeneration in mice [5, 14]. In addition, depletion of 26S proteasome activity by conditional knockout of an essential subunit of the 19S proteasome in mice leads to formation of Lewy body-like inclusions and progressive dopaminergic neurodegeneration [2].

It remains unclear whether expression of mutant α-synuclein leads to UPS dysfunction in intact dopaminergic neurons in vivo. Furthermore, the temporal relationship between accumulation of misfolded α-synuclein, UPS impairment and dopaminergic neurotoxicity in vivo is not yet elucidated. Here, we show that AAV-mediated over-expression of mutant α-synuclein in dopaminergic neurons of the SNpc in rats results in early-onset accumulation of a proteasome-targeted reporter protein which precedes behavioural dysfunction and dopaminergic neurodegeneration. These findings suggest that accumulation of misfolded α-synuclein in vivo could trigger UPS dysfunction in dopaminergic neurons, leading to progressive cellular dysfunction and eventually cell death due to proteostasis failure.

## Methods

### Animals

Adult female Sprague-Dawley rats (250–280 g; Charles River) were pair-housed in cages with wood bedding and had access to food and water ad libitum. The animal colony was maintained in a regular 12-h light/dark cycle (lights on 06:30). All procedures were approved by the University Health Network Animal Care Committee in accordance with guidelines and regulations set by the Canadian Council on Animal Care.

### Adeno-associated viruses

Adeno-associated virus (AAV) of a 1/2 serotype was used to express A53T α-synuclein (AAV-A53T) under the control of the CAG promoter, a hybrid of the chicken beta actin (CBA) promoter fused with the cytomegalovirus (CMV) immediate early enhancer sequence (2.55 × 10^12^ genomic particles (gp) per mL; GeneDetect Ltd.), as previously described [24]. An AAV1/2 vector lacking the A53T α-synuclein open reading frame was used as an empty vector control (AAV-Empty). The Ub^G76V^-GFP and TdTomato open reading frames were expressed under the control of the CAG promoter in an AAV-8 capsid serotype (AAV-Ub^G76V^-GFP and AAV-TdTomato, respectively) (1 × 10^11^ gp per mL; CyagenBiosciences). Two μL of AAV vectors were administered by stereotaxic injection into the SNpc.

### HEK 293 cell culture and transfection

HEK 293 cells were maintained at 37 °C and 5% CO_2_ in DMEM (Life Technologies) supplemented with 10% foetal bovine serum (Life Technologies) and 1% penicillin/streptomycin (Life Technologies). Cells were transfected with the AAV plasmids expressing Ub^G76V^-GFP and either empty vector or A53T α-synuclein according to manufacturer’s protocol for Lipofectamine 2000 (Thermo Fisher Scientific) 24 h prior to treatment. Control cells were treated with 10 μM MG132 for 6 h and harvested. Cells were lysed in radioimmunoprecipitation assay buffer (RIPA) and subsequently analyzed by western blot.

### Primary cortical neuron culture and transduction

Pregnant rats (E17) of the Sprague-Dawley strain were purchased from Charles River. Embryos were surgically removed from the mothers and cortices dissected in Hanks Balanced salt solution (Gibco). The meninges were removed and cells dissociated using a papain dissociation system (Worthington) before being resuspended in Neurobasal medium A supplemented with antibiotic-antimycotic solution (Gibco), L-glutamine substitute (GlutaMAX™; Gibco) and factor B27 (Gibco). Cells were plated on poly-D-lysine coated glass coverslips at a density of 5 × 10^5^ cells/well and incubated at 37 °C in 5% CO_2_ with half media changes every 3 days. Cells were transduced with AAV-A53T, AAV-Empty and AAV-Ub^G76V^-GFP 2 days post-isolation at a multiplicity of infection (MOI) of 3000. Media containing AAV vectors were removed after 72 h and cells were fixed with 4% PFA for immunofluorescence staining at 8 days post-isolation.

### Stereotaxic surgery

Animals were secured in a stereotaxic frame under isoflurane/oxygen anaesthesia and ketoprofen (5 mg/kg) analgesia. Using aseptic conditions, a 2 μL injection of viral vector was delivered into the substantia nigra pars compacta (SNpc; AP − 5.2 mm, ML +/− 2 mm, DV − 7.5 mm relative to bregma) at a rate of 0.5 μL per minute by a microinjector pump (Harvard Apparatus). For delivery of lactacystin (4 mg/mL) or sterile water vehicle, a smaller volume of 0.5 μL was delivered to the same target at a rate of 0.25 μL per minute to avoid diffusion of the compound to the contralateral midbrain.

### Cylinder test

Spontaneous forepaw use was evaluated using the cylinder test 1 day prior to stereotaxic AAV injection and at the indicated end-point. Following overnight food restriction, individual rats with right paws marked black were placed into a glass cylinder in front of two mirrors and videos recorded. An observer blinded to treatment conditions later scored videos by recording whether animals used their left or right forepaw to touch the inner glass surface on rearing. A total of 5 min of video recording was scored and a minimum of 20 total touches was required for data inclusion.

### Tissue preparation

Animals were euthanised by transcardial perfusion with heparinised saline under isoflurane/oxygen anaesthesia. Brains were then removed and placed into 4% paraformaldehyde (PFA) in Sorensen phosphate buffer for 48 h at 4 °C. A replicate cohort of animals were culled in parallel and brains snap-frozen in isopentane to provide unfixed tissue for western blotting and proteasome activity assays. PFA-fixed brains were immersed in 30% (w/v) sucrose for 72 h for cryoprotection and then in Optimal Cutting Temperature (OCT; Tissue Tek). For immunofluorescent staining, 40 μm coronal cryosections were then prepared using a sliding microtome (Leica Microsystems Inc.) and 6 series of sections stored in cryoprotectant (30% glycerol, 30% ethylene glycol, 30% distilled water and 10% phosphate buffer at pH 7.2) at − 20 °C until use. For western blotting and proteasome activity assays, unfixed midbrain tissue was isolated from snap-frozen brains by obtaining tissue punches of coronal sections on a cryostat (Leica Microsystems Inc.).

### Immunofluorescence staining of cultured cells

After fixation, cells were permeabilized with 0.2% Triton X-100 for 15 min and then incubated with primary antibodies diluted in PBS overnight at 4 °C. Following PBS wash, cells were next incubated with fluorescent secondary antibodies diluted in PBS for 1 h at room temperature. Following another PBS wash, nuclei were counterstained with DAPI (ThermoFisher) and then coverslips were mounted on slides using fluorescence mounting medium (DAKO).

### Immunofluorescence staining of cryosections

Immunofluorescence staining for GFP, α-synuclein (total or pS129) and tyrosine hydroxylase was performed by pre-treating free-floating sections with 0.1% Triton X-100 for 15 min and 1 M glycine for 30 min at room temperature. Sections were then incubated in blocking solution (1% bovine serum albumin solution, 10% normal goat serum and 0.1% Triton X-100) for 1 h. In contrast, immunofluorescence staining for polyubiquitinylated conjugates required pre-treatment in 10 mM sodium citrate buffer pH 8.5 for 30 min at 80 °C, followed by incubation in 2% non-fat dry milk in 0.3% Triton-X-100 for 45 min at room temperature. After blocking, sections were incubated with primary antibodies in blocking solution for 16 h at 4 °C (Additional file 1: Table S1). Sections were then washed in PBS and incubated with secondary antibodies diluted in PBS for 1 h in the dark at room temperature (Additional file 1: Table S2). Nuclei were counterstained with 4′,6-diamidino-2-phenylindole (DAPI; ThermoFisher) and slides mounted with fluorescence mounting medium (DAKO).

### RNA in situ hybridisation

Slide-mounted 40 μm PFA-fixed coronal sections were used to perform RNA in situ hybridisation using the RNAScope Multiplex Fluorescent Kit V2 (ACDbio). Probe sets specific for *eGFP*, *Actb* and *Th* sequences were designed by ACDbio and RNA in situ hybridisation was performed according to manufacturer’s instructions.

### Image acquisition and analysis

Confocal images of immunofluorescent staining and RNA in situ hybridisation were acquired with a Zeiss LSM880 confocal microscope equipped with 405, 488, 555 and 639 nm laser lines. All images were taken within the linear range at constant gain and pinhole settings at a resolution of 1024 × 1024 pixels. For immunofluorescent staining experiments, the whole midbrain or striatum regions were imaged using a 10X or 20X objective. Ten serial coronal sections were imaged per animal, separated by 240 μm intervals. For RNA in situ hybridisation experiments, Opal 570 (*eGFP*), Opal 690 (*Th*) and Opal 520 (*Actb*) fluorophores were visualised in 10 fields of view across the SNpc ipsilateral to the site of AAV administration using a Plan-Apochromat 63x/1.4 NA oil immersion objective. Zeiss Immersol 518F was used as imaging medium. Three consecutive coronal sections were imaged per animal.

Confocal images of immunofluorescent staining were processed using HALO software (Indica Labs), which is a validated tool for automatic quantification of neurons in brain tissue sections [18, 22, 48]. Initially, ispilateral SNpc was selected as a region of interest (ROI) by a blinded observer. Dopaminergic neurons were subsequently identified by automated detection of TH-labelled objects within this ROI. To identify TH^+^ cells with accumulation of the Ub^G76V^-GFP reporter, anti-GFP staining intensity was measured in populations of TH-labelled objects. Levels of polyubiqutinated conjugates, total α-synuclein or pS129 α-synuclein were assessed in nigral dopaminergic neurons by measuring the intensity of anti-polyubiquitin, anti-α-synuclein or anti-pS129 α-synuclein staining, respectively, in TH-labelled objects within the ROI. For cell phenotypic analyses, z-stacks encompassing the entire cell were obtained and at least 50 cells analysed for each condition.

Confocal images of RNA in situ hybridisation were processed using CellProfiler 3.0 software [7]. Dopaminergic neurons within the SNpc were identified by automated detection of TH-labelled objects. The average signal dot number per dopaminergic neuron was then calculated for each fluorophore channel separately as per manufacturer’s instruction (ACDBio SOP 45–006).

Confocal images of primary cortical neurons were processed using Immaris software (Bitplane). Z stacks were projected to give a 3D reconstruction of the field of view and mean pixel intensity per cell was calculated for Ub^G76V^-GFP signal.

### Proteasome activity assay

Homogenates of snap-frozen midbrain tissue 10% (w/v) were prepared in ice-cold proteasome assay lysis buffer (50 mM Tris-HCl, 5 mM MgCl_2_, 250 mM sucrose, 2 mM ATP at pH 7.4) by shearing with 21, 23 and 25G needles. Homogenates were then centrifuged at 13,000 x *g* for 20 min at 4 °C. Resulting supernatants were placed on ice and total protein concentrations measured using the BioRad DC Protein Assay. Samples were adjusted to 1 mg/mL final concentration in proteasome assay lysis buffer supplemented with 1 mM dithiothreitol (DTT) and used for kinetic or in-gel native PAGE assays of proteasome activity. For kinetic proteasome hydrolysis assays, the rate of hydrolysis was measured by incubating 10 μg of sample with 100 μM fluorogenic peptide substrate in 100 μL of proteasome assay reaction buffer (50 mM Tris-HCl, 5 mM MgCl_2_, 1 mM DTT, 2 mM ATP at pH 7.4). Chymotrypsin-like activity was determined using the substrate Suc-LLVY aminomethylcoumarin (AMC; Enzo Life Sciences); caspase-like activity was determined using the substrate Ac-Nle-Pro-Nle-Asp-AMC (Bachem); and, trypsin-like activity was determined using the substrate Boc-Leu-Arg-Arg-AMC (Enzo Life Sciences). Samples were incubated for 1 h at 37 °C and the release of AMC measured at 1 min intervals in a BMG Labtech CLARIOstar 96-well plate reader (360 nm excitation; 465 nm emission). All assays were performed in triplicate. Background activity caused by non-proteasome degradation was measured by addition of 5 μM epoxomicin (Enzo Life Sciences) for 30 min at 37 °C.

For in-gel native PAGE assay of proteasome activity, samples were loaded on to a 3–5% native gradient gel incubated at 4 °C and run for 200 min at 150 V as previously described by Myeku et al. [35]. Activity of the 26S proteasome was then measured by incubating the gel in 400 μM Suc-LLVY-AMC (Enzo Life Sciences) diluted in a proteasome activity buffer containing 50 mM Tris-HCl at pH 7.4, 5 mM MgCl_2_, 10% glycerol (v/v), 1 mM ATP and 1 mM DTT at 37 °C for 10 min. Proteasome bands were visualised by placing the gel on a UV transilluminator with proteasome activity buffer. The gel was subsequently transferred onto PVDF membrane for 2 h at 110 mA at 4 °C to allow subsequent normalisation of in-gel activity to proteasome subunit levels measured by western blot.

### Western blot analysis

Tissue homogenates 10% (w/v) were prepared by homogenizing snap-frozen midbrain tissue in ice-cold tissue lysis buffer (100 mM Tris-HCl, 100 mM NaCl, 1% Triton X-100, 10 mM EDTA at pH 7.4) supplemented with cOmplete Mini protease and PhosSTOP phosphatase inhibitors (Roche) and centrifuged at 13,000 x g for 20 min at 4 °C. Supernatants were then stored on ice and total protein concentrations measured using the BioRad DC Protein Assay kit. Samples were adjusted to 1 mg/mL total protein concentration with lysis buffer and SDS-PAGE loading buffer, before being incubated at 96 °C for 5 min. Once cool, samples were resolved on 4–15% SDS-PAGE gels and probed with antibodies to β5 (PW8895; Enzo Life Sciences), Rpt6 (PW9265; Enzo Life Sciences) and α/β tubulin (2148S; Cell Signaling). Immunoblots were developed using HRP-conjugated secondary antibodies and chemiluminescent detection methods.

### Data analysis

For in vitro studies, a minimum of 3 independent experiments were carried out and 10 cells were analysed per experiment. For in vivo studies, 3–14 animals per group were analyzed. Data are presented as mean or percentage mean +/− SEM, unless otherwise specified. GraphPad Prism version 7 software (GraphPad Software Inc.) was used to perform all data analysis.

For the cylinder test, the percentage asymmetry in forepaw use was calculated by the following equation: (% right paw use - % left paw use)/(% right paw use + % left paw use) × 100. For each individual animal, percentage asymmetry in forepaw use was expressed relative to baseline measurements from 1 day prior to stereotaxic AAV injection. Data were then analysed by one-way ANOVA with Tukey post-hoc tests as we have previously described [23, 24].

For immunofluorescent staining experiments, an intensity value of two standard deviations greater than the population mean for AAV-Empty control rats at each time point was set as a threshold for classifying a cell as GFP-positive. The density of GFP-positive dopaminergic neurons and polyubiquitinated conjugate level of dopaminergic neurons were each compared between treatment groups using one-way ANOVA with Tukey post-hoc tests. For RNA in situ hybridisation experiments, relative *Ub*^*G76V*^*-GFP* mRNA levels were calculated by normalising the mean *Ub*^*G76V*^*-GFP* staining intensity to the mean staining intensity of the *Actb* control transcript and subsequently comparing AAV-Empty, AAV-TdTomato and AAV-A53T α-synuclein groups using a one-way ANOVA. To assess the relationship between A53T α-synuclein expression and polyubiquitinated conjugate levels in nigral dopaminergic neurons, Spearman correlations were calculated for individual animals in the AAV-A53T α-synuclein group.

In the kinetic proteasome activity assay, catalytic activity was calculated by measuring the mean fluorescence generated in the linear reaction phase across triplicate wells. To calculate specific proteasome catalytic activities, mean fluorescence values of epoxomicin-treated controls were subtracted from each sample and subsequently expressed as a percentage of the control tissue mean. For the in-gel native PAGE proteasome activity assay, chymotrypsin-like activity was normalised to proteasome subunit levels measured by western blot densitometry and again expressed as a percentage of the control tissue mean. For both proteasome assays, catalytic activity in ipsilateral (AAV-A53T) and contralateral (AAV-Empty or AAV-TdTomato) midbrain tissue was compared using paired t-tests. For proteasome subunit quantification experiments, scanned immunoblots were converted to grey-scale images and signal intensity compared by one-way ANOVA. Levels of total α-synuclein and pS129 α-synuclein immunofluorescent staining in Ub^G76V^GFP^−^ and Ub^G76V^GFP^+^ dopaminergic neurons were compared using two-tailed Student’s t tests.

## Results

### α-Synuclein expression is associated with Ub^G76V^-GFP reporter accumulation in neurons in vitro

**Fig. 1:**
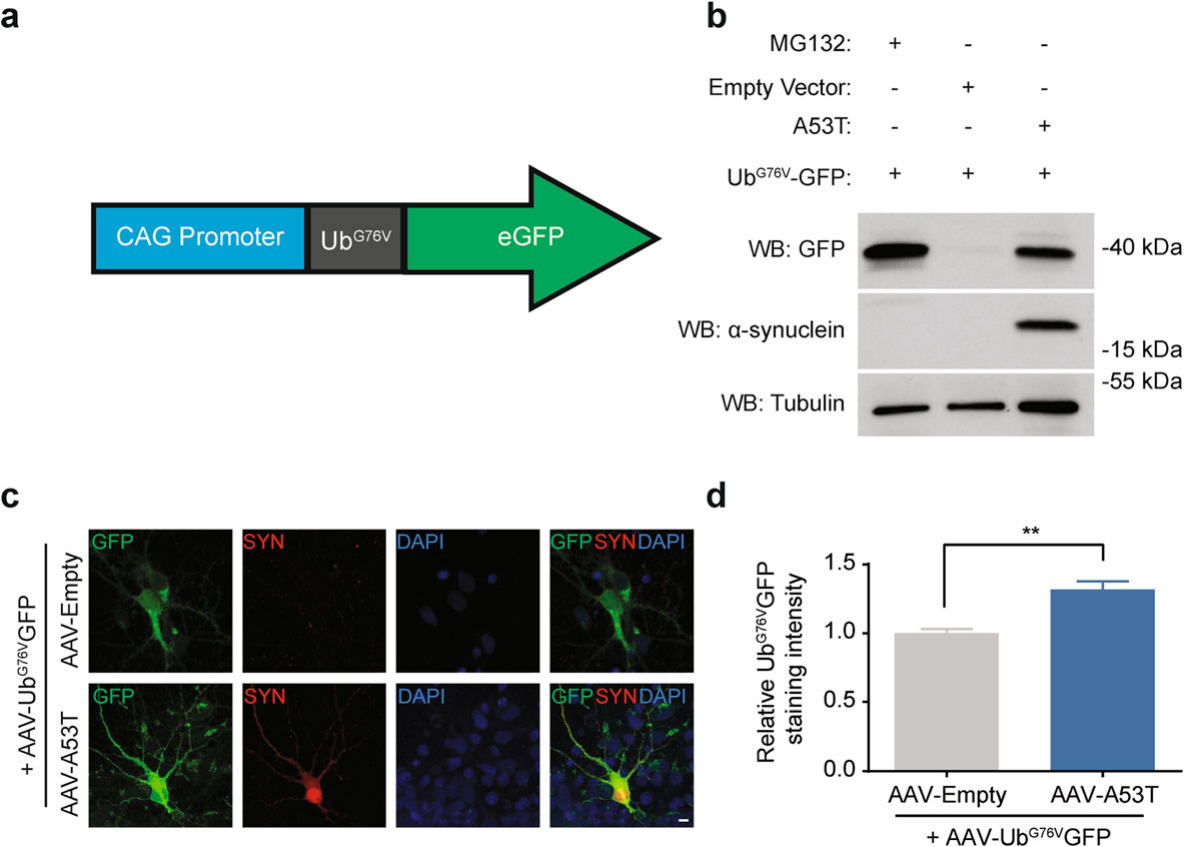
α-Synuclein expression is associated with increased levels of Ub^G76V^-GFP in HEK cells and in primary cortical neurons. **a** Schematic of the UPS reporter. Expression of Ub^G76V^-conjugated GFP is driven by the CAG promoter, a hybrid of the CBA and CMV promoters. **b** Representative western blot demonstrating Ub^G76V^-GFP accumulation in response to MG132 treatment and A53T α-synuclein over-expression in HEK293 cells. **c** Primary rat cortical neurons were infected with AAV-Ub^G76V^-GFP and either AAV-Empty or AAV-A53T at 3000 MOI at day 2 in vitro and fixed 6 days later. Representative images of anti-GFP (*green)* and anti-α-synuclein (*red)* immunofluorescent staining with DAPI nuclear stain (*blue)* are shown. *Scale bar* 5 μm. **d** Cultures infected with AAV-A53T α-synuclein display a 31% increase in mean neuronal GFP staining intensity relative to those infected with AAV-Empty vector control (**p < 0.01; *n* = 3 independent experiments, analyzing a minimum of 10 cells per condition)

To investigate the effect of α-synuclein expression on UPS activity in neurons, we sought to develop a viral UPS reporter which could be used to label neurons experiencing UPS failure. Ub^G76V^-GFP is a reporter protein with a ubiquitin fusion degradation signal consisting of an N-terminally linked ubiquitin moiety which accepts polyubiquitin chains linked through Lys48 and Lys29 linkages [28] (Fig. 1a). In the context of UPS impairment, reduction in clearance of polyubiquitinated substrates by the 26S proteasome results in accumulation of Ub^G76V^-GFP, which can be detected by native fluorescence or immunofluorescent staining with anti-GFP antibodies [28]. To test our constructs, we transfected HEK293 cells with plasmids expressing Ub^G76V^-GFP and mutant A53T α-synuclein or empty vector control. We found that pharmacological proteasome inhibition by MG132 and A53T over-expression resulted in Ub^G76V^-GFP accumulation (Fig. 1b). We next generated adeno-associated viral vectors (AAV) for expression of Ub^G76V^-GFP (AAV-Ub^G76V^-GFP) and human A53T α-synuclein (AAV-A53T α-synuclein) in neurons. Co-infection of primary rat cortical neurons with AAV-Ub^G76V^-GFP and AAV-A53T α-synuclein resulted in a 31% increase in mean neuronal GFP staining intensity relative to co-infection with a control AAV containing an empty vector (AAV-Empty) (Fig. 1c, d). This finding of increased levels of Ub^G76V^-GFP suggests that neuronal expression of A53T α-synuclein is associated with UPS dysfunction in neurons in vitro.

### Proteasome inhibition in nigral dopaminergic neurons is associated with Ub^G76V^-GFP reporter accumulation in vivo

To validate AAV-Ub^G76V^-GFP as a tool to measure neuronal UPS activity in vivo, the vector was administered by bilateral stereotaxic injection into the substantia nigra pars compacta (SNpc) of adult wild-type rats and, at 3 weeks post-injection (wpi), proteasome inhibition was induced by unilateral SNpc infusion of lactacystin. Relative to infusion of vehicle control to the contralateral SNpc, lactacystin infusion was associated with a marked increase in the number of GFP-positive dopaminergic neurons in the SNpc (Additional file 1: Figure S1). Following vehicle administration, a small cluster of GFP-positive dopaminergic neurons was observed in close proximity to the needle track. This may have reflected high viral genome copy numbers of AAV-Ub^G76V^-GFP in cells surrounding the injection target, rather than localised UPS dysfunction.

**Fig. 2:**
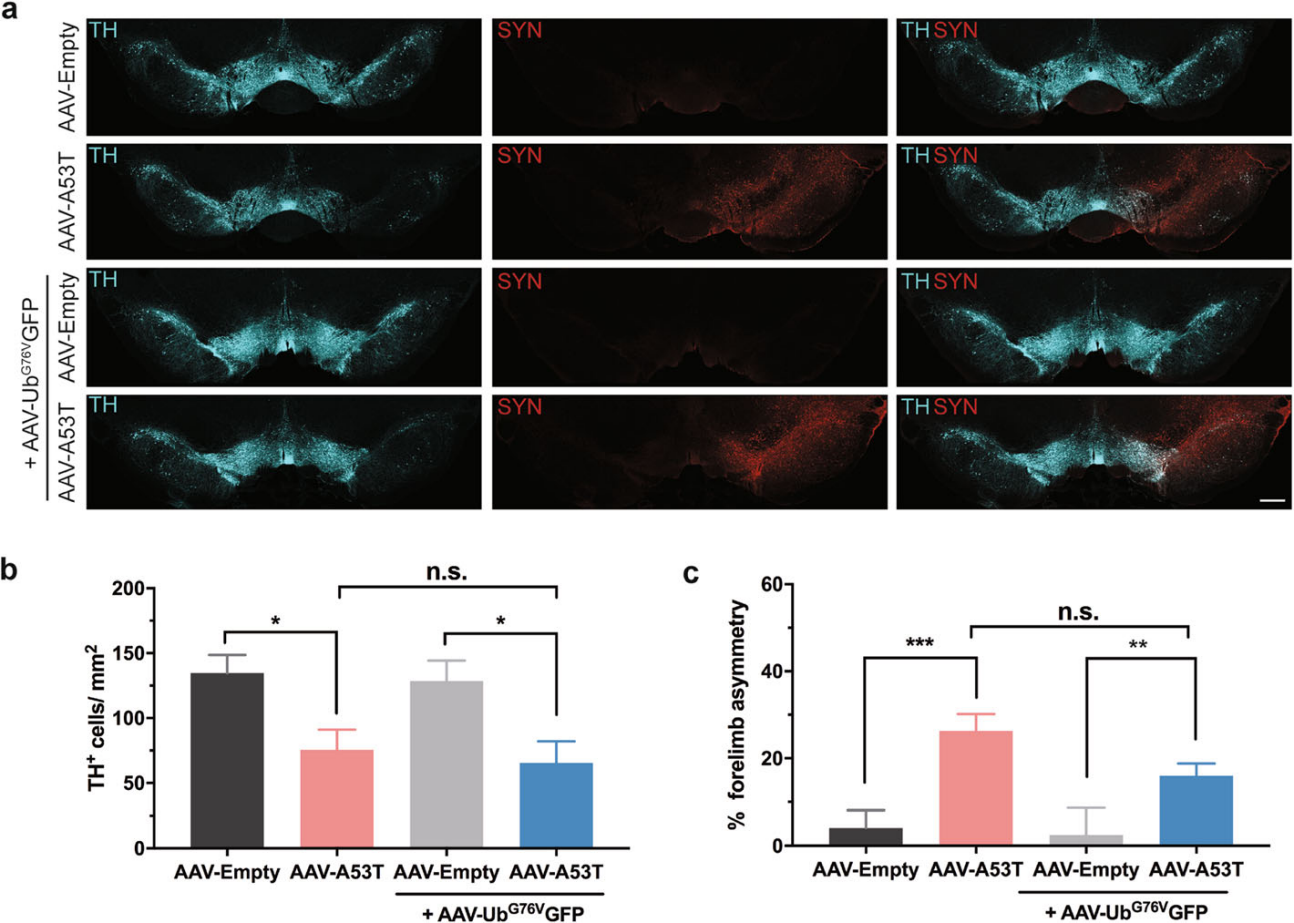
Co-expression of AAV-Ub^G76V^-GFP viral UPS reporter does not modify A53T α-synuclein expression, dopaminergic neuronal loss or forelimb asymmetry in AAV-A53T treated rats. Adult wild-type rats received unilateral stereotaxic injection of AAV-A53T or AAV-Empty, either alone or in combination with AAV-Ub^G76V^-GFP, into the right SNpc. Animals were culled 3 weeks following virus administration. **a** Representative images of anti-tyrosine hydroxylase (TH; *cyan)* and anti-α-synuclein (SYN; *red*) immunofluorescent staining of coronal cryosections show similar patterns of A53T α-synuclein expression and dopaminergic neuronal loss in ipsilateral SNpc of animals injected with AAV-A53T and AAV-A53T/AAV-Ub^G76V^-GFP. *Scale bar* 500 μm. **b** Quantification of TH-labelled cells in SNpc ipsilateral to site of AAV injection. A significant reduction in the density of dopaminergic neurons is observed at 3 wpi with no significant difference (n.s.) between AAV-A53T and AAV-A53T/AAV-Ub^G76V^-GFP-treated groups. Data are mean +/− SEM (*p < 0.05; one-way ANOVA with Tukey post-hoc tests; *n* = 6–8 per group). **c** Measurement of forelimb asymmetry in the cylinder test at 3 wpi. Rats injected with AAV-A53T display significant asymmetry in forelimb use with no effect of co-administration of AAV-Ub^G76V^-GFP. Data are percentage mean +/−SEM (*p < 0.05; **p < 0.01; ***p < 0.001; one-way ANOVA with Tukey post-hoc tests; *n* = 7–15 per group)

Targeted over-expression of α-synuclein in the SNpc is associated with loss of dopaminergic neurons [23, 24]. To confirm that co-expression of the Ub^G76V^-GFP reporter does not exacerbate α-synuclein-mediated nigral degeneration, we examined the effect of co-injecting AAV-Ub^G76V^-GFP with AAV-A53T α-synuclein into rat SNpc on the severity of dopaminergic neuronal loss and forelimb asymmetry (Fig. 2). Three weeks following virus administration, α-synuclein expression in the SNpc was comparable between rats that received AAV-Ub^G76V^-GFP with AAV-A53T α-synuclein and rats that received AAV-Ub^G76V^-GFP with AAV-Empty control (Fig. 2a). We observed a 44% reduction in the density of nigral TH-immunoreactive neurons in animals that received AAV-A53T α-synuclein relative to animals that were injected with AAV-Empty control, with no significant effect of AAV-Ub^G76V^-GFP co-administration (Fig. 2a, b). Similarly, 3 weeks following AAV-A53T α-synuclein injection into the SNpc, AAV-Ub^G76V^-GFP did not exacerbate forelimb asymmetry in the cylinder test, a sensitive measure of motor impairment due to nigrostriatal dysfunction in this model [39] (Fig. 2c). Thus, we conclude AAV- Ub^G76V^-GFP can be used to detect functional impairment of the UPS in nigral dopaminergic neurons, without any significant confounding neurotoxicity arising from expression of the Ub^G76V^-GFP reporter protein itself.

### Ub^G76V^-GFP reporter accumulates early following α-synuclein expression in nigral dopaminergic neurons in vivo

**Fig. 3:**
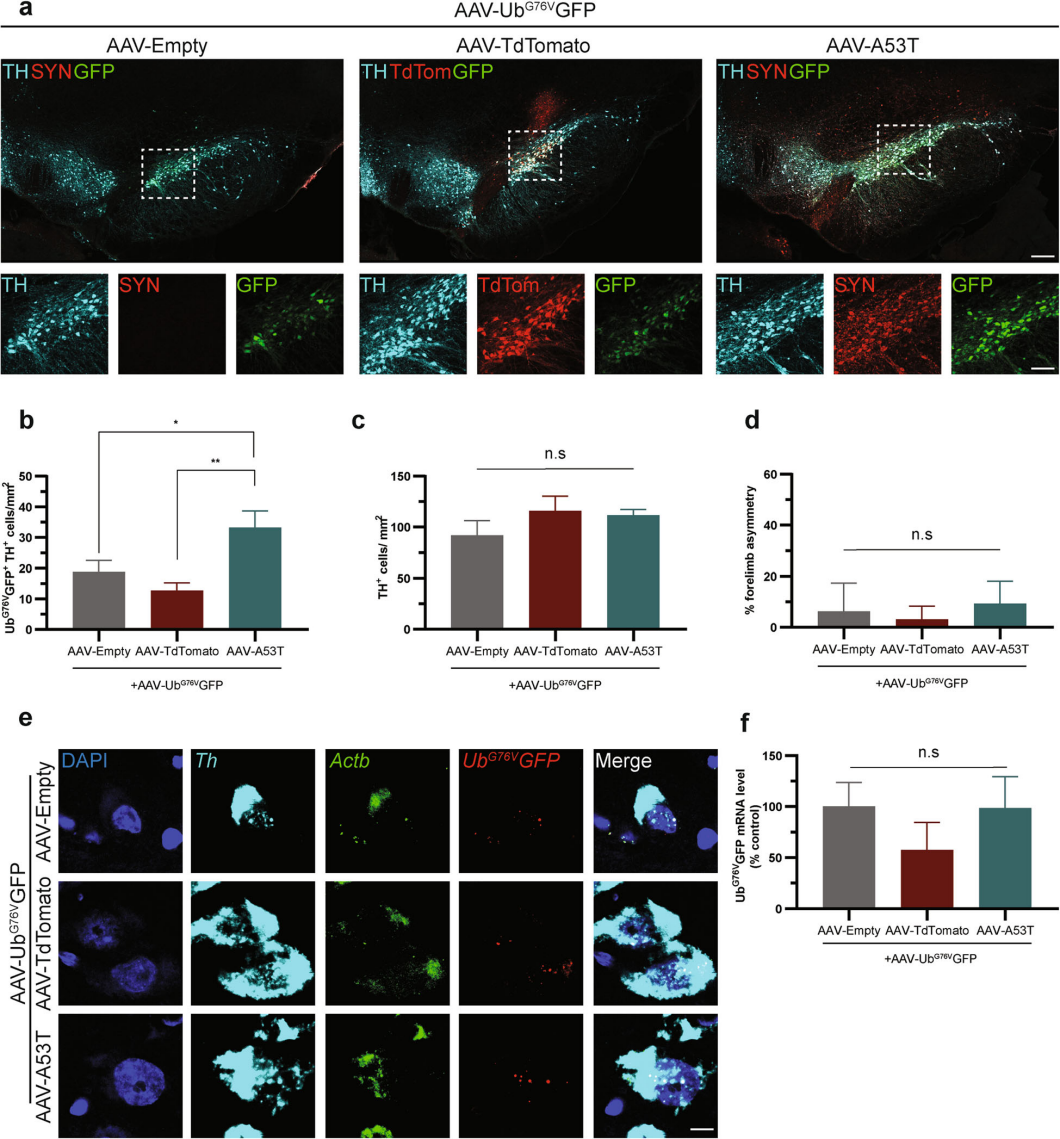
A53T α-synuclein over-expression in nigral dopaminergic neurons is associated with early-onset of accumulation of the Ub^G76V^-GFP reporter. Adult wild-type rats received unilateral stereotaxic co-injection of AAV-Ub^G76V^-GFP with AAV-A53T, AAV-Empty or AAV-TdTomato into the right SNpc. Animals were culled at 1 wpi (*n* = 12–14 per group). **a** Comparison of immunofluorescent staining with anti-tyrosine hydroxylase (TH; *cyan)*, anti-GFP (GFP; *green*), anti-α-synuclein (SYN; *red*) antibodies or native TdTomato fluorescence (TdTom; *red*) in SNpc at 1 wpi. *Scale bar* 500 μm (inset 50 μm). **b** Quantification of Ub^G76V^-GFP^+^/TH^+^ neurons per mm^2^ reveals accumulation of the UPS reporter in nigral dopaminergic neurons overexpressing A53T α-synuclein, relative to both empty vector and TdTomato controls at 1 wpi. **c** Quantification of TH^+^ neurons per mm^2^ reveals no difference in the density of nigral dopaminergic neurons 1 week after AAV injection into the ipsilateral SNpc. **d** Measurement of forelimb use by the cylinder test at 1 wpi shows no significant difference in forelimb asymmetry between groups. Data are percentage mean +/−SEM (one-way ANOVA; n = 7–8 per group). **e** Representative images from multiplex RNA in situ hybridisation experiment to compare Ub^G76V^-GFP (red) and Actb (green) transcript levels in Th-labelled neurons (cyan) 1 week following stereotaxic administration of AAV-Ub^G76V^-GFP/AAV-Empty, AAV-Ub^G76V^-GFP/AAV-TdTomato or AAV-Ub^G76V^-GFP/AAV-A53T into the right SNpc. Nuclei were counterstained with DAPI (blue). Scale bar 5 μm. **f** Quantification of Ub^G76V^-GFP mRNA levels normalised to beta-actin transcript levels in Th-labelled neurons 1 week following AAV injection into ipsilateral SNpc reveals no significant difference in expression of the Ub^G76V^-GFP reporter transgene between experimental groups. Data are percentage mean +/− SEM (*p* = 0.5336; one-way ANOVA; *n* = 4 per group)

To determine the functional status of the UPS at an early timepoint following A53T α-synuclein over-expression in rat SNpc, we measured Ub^G76V^-GFP reporter levels by anti-GFP immunofluorescent staining in the SNpc following timed culls at 1 wpi. Animals received co-injection of AAV-Ub^G76V^-GFP with either AAV-A53T α-synuclein, AAV-Empty control or AAV-TdTomato, with the latter acting as a non-disease associated protein over-expression control. One week following AAV-A53T α-synuclein administration, there was a 77% increase in GFP-positive dopaminergic neurons compared with AAV-Empty control (Fig. 3a, b). In contrast, AAV-TdTomato administration was not associated with increased numbers of GFP-positive dopaminergic neurons compared with AAV-Empty control (Fig. 3a, b). To determine whether accumulation of the Ub^G76V^-GFP reporter protein was a result of impaired protein degradation rather than increased viral transduction or protein synthesis, we measured *Ub*^*G76V*^*-GFP* transcript levels by RNA in situ hybridization (Fig. 3e). No differences in *Ub*^*G76V*^*-GFP* transcript levels were observed 1 week following co-injection of AAV-Ub^G76V^-GFP with either AAV-A53T α-synuclein, AAV-Empty or AAV-TdTomato into the SNpc (Fig. 3f). Thus, the increase in Ub^G76V^-GFP reporter protein levels in animals expressing A53T α-synuclein is due to impaired reporter degradation, rather than increased expression or stabilization of the *Ub*^*G76V*^*-GFP* transcript. Notably, the accumulation of Ub^G76V^-GFP following ΑAV-A53T α-synuclein expression preceded the onset of forelimb asymmetry in the cylinder test (Fig. 3d) and occurred prior to any detectable dopaminergic neuronal loss in the SNpc (Fig. 3a, c).

### Ub^G76V^-GFP reporter does not accumulate in striatum following α-synuclein expression in nigral dopaminergic neurons

**Fig. 4:**
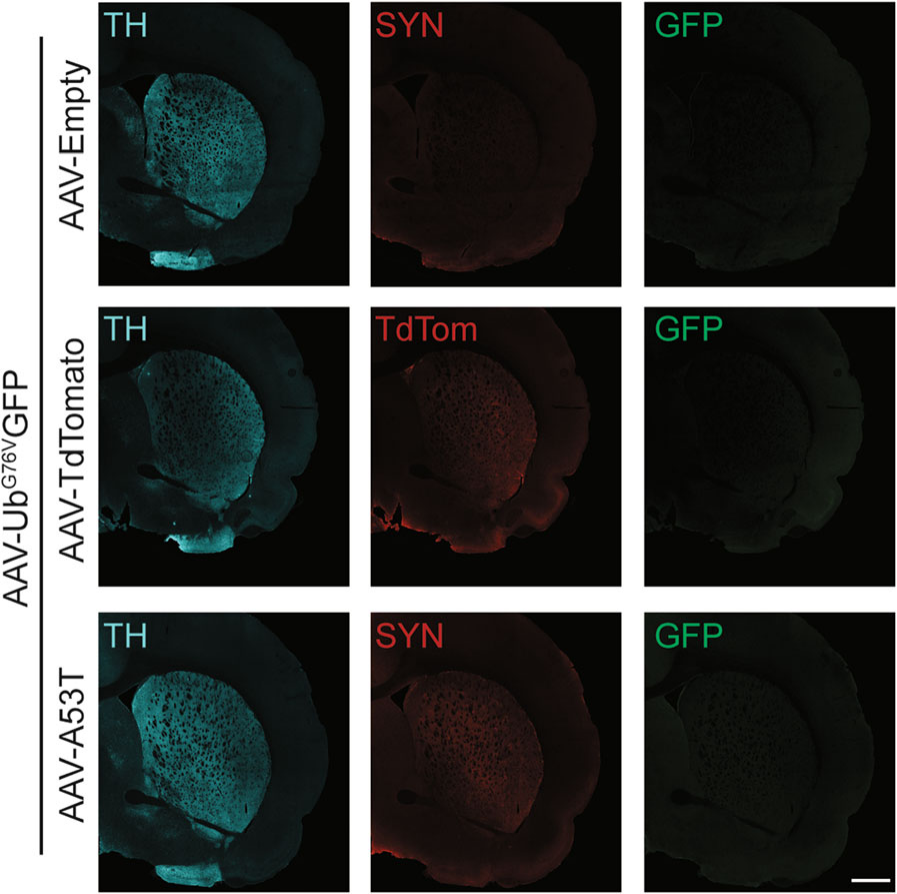
Ub^G76V^-GFP reporter does not accumulate in the striatal projections of the SNpc following 1 week of A53T α-synuclein over-expression. Adult wild-type rats received unilateral stereotaxic co-injection of AAV-Ub^G76V^-GFP with AAV-A53T, AAV-Empty or AAV-TdTomato into the right SNpc. Animals were culled at 1 wpi. Representative immunofluorescent staining of ipsilateral striatum with anti-tyrosine hydroxylase (TH; *cyan)*, anti-GFP (GFP; *green*), anti-α-synuclein (SYN; *red*) antibodies or native TdTomato fluorescence (TdTom; *red*) at 1 wpi is shown (*n* = 3 per group). No evidence of Ub^G76V^-GFP reporter accumulation in striatal terminals is observed, despite widespread expression of A53T and TdTomato transgenes. *Scale bar* 500 μm

We next examined whether this early-onset UPS dysfunction observed following A53T α-synuclein expression in nigral dopaminergic neurons is accompanied by UPS impairment in their striatal projections. Specifically, we examined Ub^G76V^-GFP levels in TH-immunoreactive neurites in striatal sections. We observed no evidence of GFP immunoreactivity in the striatum of animals who received nigral injection of AAV-Ub^G76V^-GFP with either AAV-A53T α-synuclein or AAV-TdTomato at 1 wpi, despite widespread transgene expression (Fig. 4). We therefore conclude that A53T α-synuclein over-expression at an early timepoint is associated with UPS dysfunction that predominantly affects the soma of nigral dopaminergic neurons with relative sparing of downstream striatal projections.

### α-Synuclein over-expression in substantia nigra is associated with inhibition of proteasome catalytic activity

**Fig. 5:**
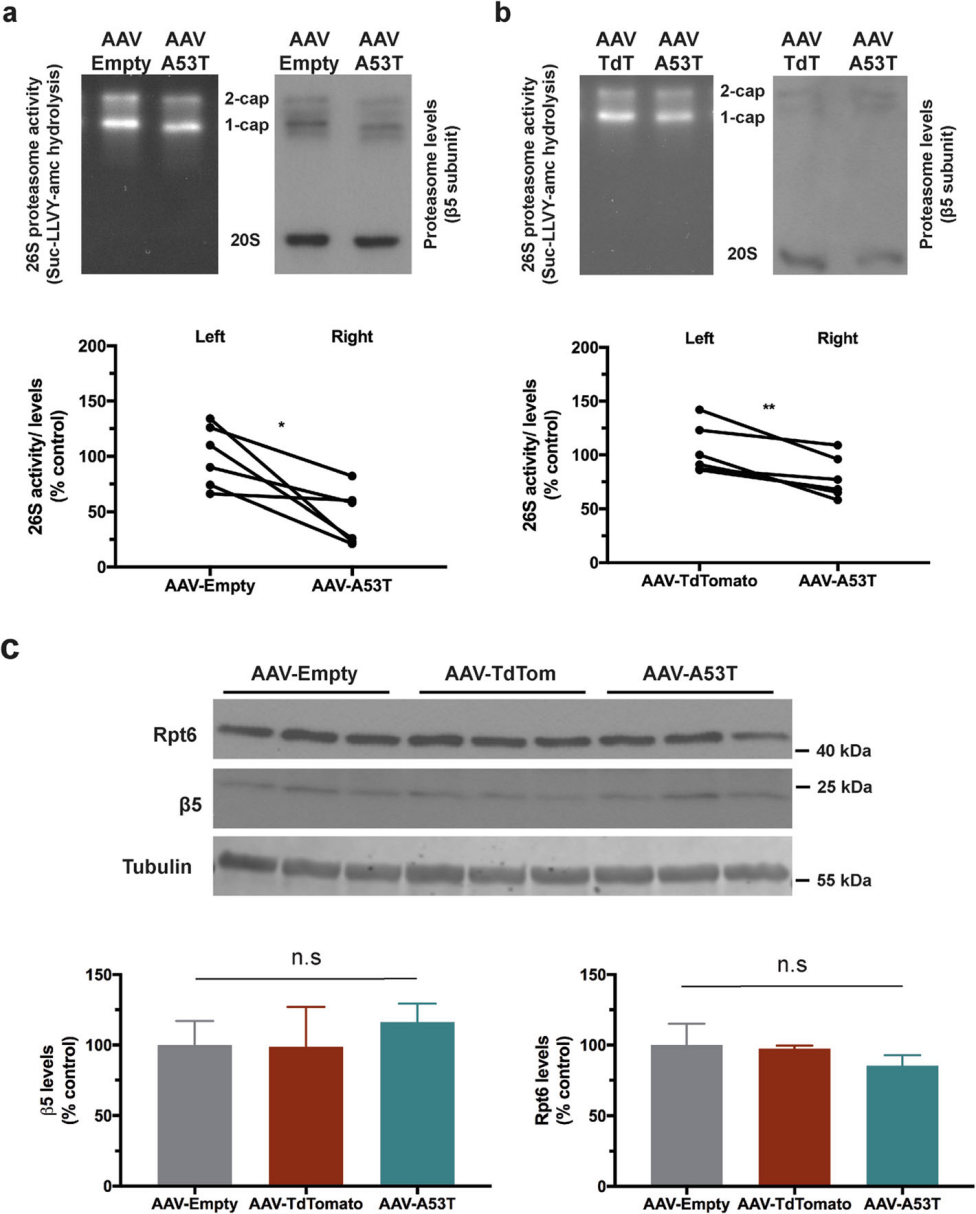
A53T α-synuclein over-expression is associated with inhibition of 26S proteasome catalytic activity. Adult wild-type rats received stereotaxic injection of AAV-Empty or AAV-TdTomato (TdT) into the left SNpc and AAV-A53T into the right SNpc. Animals were culled at 1 wpi before isolation of left and right midbrain tissue. **a-b** Native PAGE assay of 26S proteasome activity and subsequent immunoblotting of β5 proteasome subunit levels were compared between left (AAV-Empty (**a**) or AAV-TdTomato (**b**)) and right (AAV-A53T) midbrain samples. Quantification by densitometry and normalisation to proteasome subunit levels revealed that A53T α-synuclein over-expression was associated with significant inhibition of 26S catalytic activity. Data are individual percentage values expressed relative to mean of control group (*p < 0.05, **p < 0.01; paired t-test; n = 6 per group). **c** Representative immunoblot comparing Rpt6 and β5 proteasome subunit levels in midbrain samples expressing AAV-Empty, AAV-TdTomato or AAV-A53T. Quantification by densitometry and normalisation to tubulin loading control revealed no significant change in expression level of either subunit. Data are percentage mean expressed relative to mean of AAV-empty control group +/− SEM (β5 subunit - *p* = 0.8034, Rpt6 subunit- *p* = 0.4902; one-way ANOVA; n = 4–6 per group)

To determine whether the observed UPS dysfunction could be attributed to inhibition of proteasome catalytic activity, we compared chymotrypsin-like activity of the 26S proteasome 1 week following injection of AAV-Empty or AAV-TdTomato (to the left SNpc) and AAV-A53T α-synuclein (to the right SNpc). We measured chymotrypsin-like activity by first resolving homogenized midbrain tissue by native PAGE and then measuring in-gel hydrolysis of Suc-LLVY-7-amino-4-methylcoumarin (AMC), a fluorogenic peptide substrate of the 20S proteasome. Cleavage of this AMC-conjugated peptide by the chymotrypsin-like site releases the AMC fluorophore which can be measured under UV transillumination. To control for differences in levels of 26S proteasomes between native PAGE samples, activity levels were normalized to levels of the β5 subunit of the 20S proteasome measured by subsequent immunoblot. A 52% reduction in chymotrypsin-like activity was observed in midbrain tissue 1 week following AAV-A53T α-synuclein administration relative to contralateral midbrain tissue injected with AAV-Empty (Fig. 5a); a 25% reduction was observed relative to midbrain tissue injected with AAV-TdTomato (Fig. 5b).

The Suc-LLVY-AMC peptide is not a substrate specific to the 20S proteasome but can be cleaved by other chymotrypsin-like proteases and calpains [17]. Therefore, we measured catalytic turnover of fluorogenic peptide substrates specific to the chymotrypsin-, caspase- and trypsin-like sites of the proteasome over time in a kinetic plate-based assay. Similar to results obtained with the in-gel assay, a 46% reduction in chymotrypsin-like activity was measured in midbrain tissue expressing AAV-A53T α-synuclein relative to contralateral tissue injected with AAV-Empty (Additional file 1: Figure S2a). In addition, a 46% reduction in caspase-like activity and 59% inhibition of trypsin-like activity was observed 1 week following A53T α-synuclein expression, relative to AAV-Empty control (Additional file 1: Figure S2b, c). This impairment in proteasome peptidase activity was not due to a reduction in the size of the proteasome pool, as there was no difference in levels of the proteasome subunits Rpt6 or β5 between midbrain tissue expressing AAV-A53T α-synuclein, AAV-Empty or AAV-TdTomato (Fig. 5c). Collectively, these results indicate that A53T α-synuclein expression in midbrain is associated with early-onset inhibition of 26S proteasome catalytic activity.

### Polyubiquitinated conjugates accumulate in nigral dopaminergic neurons expressing α-synuclein

**Fig. 6:**
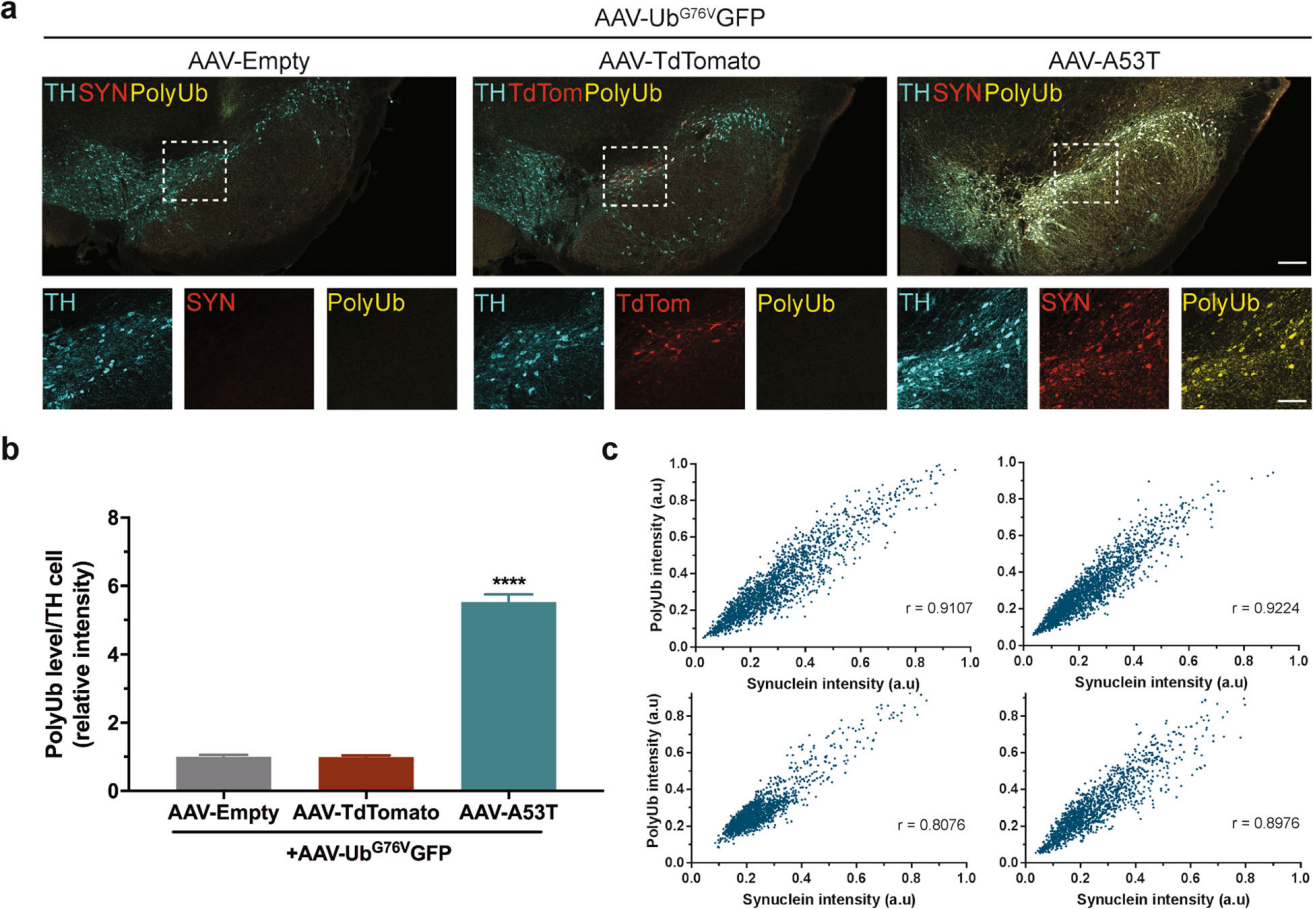
A53T α-synuclein over-expression in nigral dopaminergic neurons is associated with accumulation of polyubiquitinated conjugates. Adult wild-type rats received unilateral stereotaxic co-injection of AAV-Ub^G76V^-GFP with AAV-A53T, AAV-Empty or AAV-TdTomato into the right SNpc. Animals were culled at 1 wpi. **a** Comparison of immunofluorescent staining with anti-tyrosine hydroxylase (TH; *cyan)*, anti-polyubiquitinated conjugate (PolyUb; *yellow*), anti-α-synuclein (SYN; *red*) antibodies or native TdTomato fluorescence (TdTom; *red*) in SNpc at 1 wpi. *Scale bar* 500 μm (inset 50 μm). **b** Quantification of mean anti-polyubiquinated conjugate staining intensity in TH^+^ neurons of SNpc 1 week following ipsilateral AAV injection. A significant increase in levels of polyubiquitinated conjugates is observed in nigral dopaminergic neurons of animals expressing AAV-A53T α-synuclein/AAV-Ub^G76V^-GFP, relative to AAV-Empty/AAV-Ub^G76V^-GFP and AAV-TdTomato/AAV-Ub^G76V^-GFP control groups (****p < 0.0001; one-way ANOVA with post-hoc Tukey tests*; n* = 3–5 per group). **c** Representative scatter plots displaying relationship between anti-synuclein and anti-polyubiquitinated conjugate staining intensities in individual TH^+^ neurons in SNpc 1 week after ipsilateral AAV-A53T α-synuclein/AAV-Ub^G76V^-GFP administration. A significant positive correlation between levels of A53T α-synuclein and polyubiquitinated conjugates in nigral dopaminergic neurons is observed (Spearman correlation; individual r values as displayed for n = 4 animals; *p* < 0.0001)

To assess whether the inhibition of 26S proteasome catalytic activity due to α-synuclein over-expression is associated with impaired substrate clearance, we measured levels of polyubiquitinated conjugates in nigral dopaminergic neurons 1 week following co-administration of AAV-Ub^G76V^-GFP with either AAV-A53T α-synuclein, AAV-Empty control or AAV-TdTomato. A 5.5-fold increase in levels of polyubiquitinated conjugates was observed in animals expressing AAV-A53T α-synuclein, relative to both AAV-Empty and AAV-TdTomato control groups (Fig. 6a, b). At the single-cell level, there was a strong correlation between anti-synuclein and anti-polyubiquitin immunofluorescent staining intensity (Fig. 6c), suggesting a dose-response relationship between levels of misfolded protein in dopaminergic neurons and the extent of proteostasis network dysfunction.

### Impairment of the UPS is associated with accumulation of phosphorylated α-synuclein in vivo

**Fig. 7:**
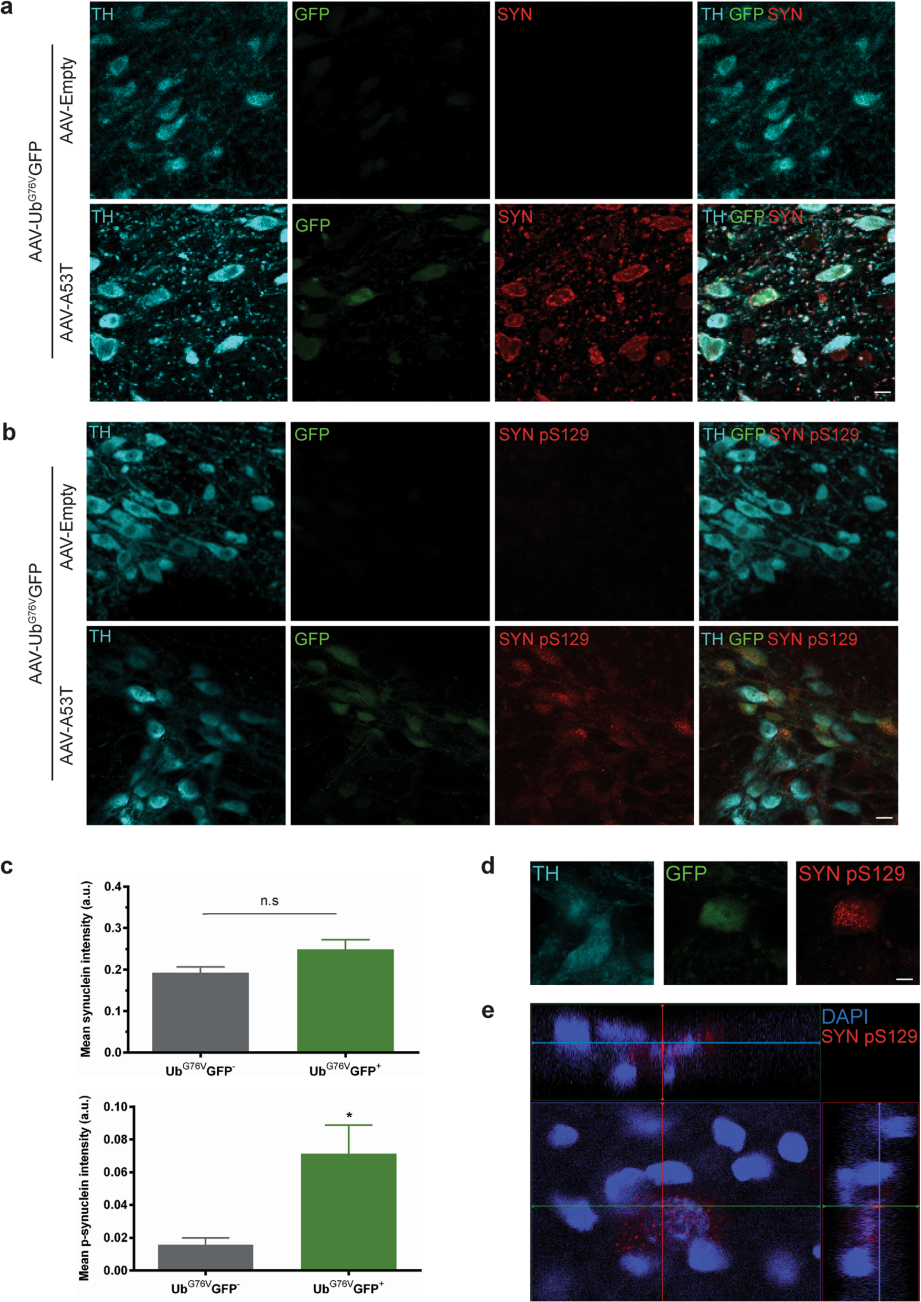
UPS impairment in nigral dopaminergic neurons is associated with perinuclear accumulation of phosphorylated α-synuclein species. Adult wild-type rats received unilateral stereotaxic co-injection of AAV-Ub^G76V^-GFP with AAV-A53T or AAV-Empty into the right SNpc. Animals were culled at 1 wpi. **a-b** Immunofluorescent staining with anti-tyrosine hydroxylase (TH; *cyan)*, anti-GFP (GFP; *green*) and either **(a)** anti-α-synuclein (SYN; *red*) or **(b)** anti-pS129 α-synuclein (SYNpS129; *red*) antibodies in SNpc at 1 wpi of AAV-A53T/AAV-Ub^G76V^-GFP or AAV-Empty/AAV-Ub^G76V^-GFP. *Scale bars* 20 μm. **c** Quantification of mean total α-synuclein and mean pS129 α-synuclein staining intensities in TH-labelled neurons classified as Ub^G76V^-GFP^−^ or Ub^G76V^-GFP^+^ in ipsilateral SNpc 1wpi of AAV-A53T/AAV-Ub^G76V^-GFP. Dopaminergic neurons with elevated levels of Ub^G76V^-GFP indicative of UPS dysfunction have elevated levels of phosphorylated α-synuclein species, independent of a significant change in total α-synuclein levels. Data are mean +/− SEM (*p < 0.05*;* two-tailed Student’s *t* test; *n* = 3 per group). **d** Representative high magnification image of TH-labelled neuron with elevated levels of Ub^G76V^-GFP reporter reveals presence of punctate pS129 α-synuclein staining. Punctate pS129 α-synuclein staining is not observed in neighbouring TH-labelled neuron with basal Ub^G76V^-GFP levels. *Scale bar* 5 μm. **e** Orthogonal z-stack view of the Ub^G76V^-GFP^+^ TH-labelled neuron shown in **(d)** with anti-pS129 α-synuclein staining (SYNpS129; *red*) and DAPI nuclear counterstain shown. Puncta of phosphorylated α-synuclein species have a perinuclear location

α-Synuclein itself is a known substrate of the UPS [4]. To test whether UPS impairment could contribute to neurotoxicity through a failure to degrade α-synuclein, we examined nigral dopaminergic neurons with and without UPS dysfunction at the single cell level and compared levels of total and phosphorylated α-synuclein. Specifically, we examined α-synuclein phosphorylated at serine 129 (pS129) (Fig. 7a, b) since the pathological protein aggregates in PD are composed primarily of this phosphorylated form of α-synuclein, and pS129 α-synuclein is known to accumulate in the presence of proteasome impairment in vitro [9]. Despite similar levels of total anti-α-synuclein staining intensity in the presence and absence of UPS dysfunction, GFP-positive nigral dopaminergic neurons displayed a significant increase in levels of pS129 α-synuclein (Fig. 7c). Orthogonal cross-section views of confocal z-stacks revealed dense pS129 α-synuclein staining in perinuclear puncta of GFP-positive dopaminergic neurons (Fig. 7d, e). This distinct staining phenotype was not observed in neighbouring GFP-negative dopaminergic neurons, suggesting there could be accumulation and altered trafficking of pS129 α-synuclein in the context of UPS impairment. Therefore, UPS impairment by α-synuclein may further enhance neurotoxicity through accumulation of pathologically phosphorylated α-synuclein protein aggregates.

## Discussion

In the present study, we identify early-onset functional impairment of the UPS following expression of A53T α-synuclein in dopaminergic neurons of the SNpc. We demonstrate that this UPS dysfunction can be attributed, at least in part, to catalytic impairment of the 26S proteasome. The onset of UPS dysfunction preceded the development of forelimb asymmetry and neuronal loss, suggesting it could be an important earlier mediator of PD pathogenesis. Consistent with this hypothesis, the observed impairment of UPS activity was accompanied by marked accumulation of polyubiquitinated conjugates and pS129 α-synuclein in affected neurons, suggesting a loss of cellular proteostasis. Taken together, these findings indicate that onset of UPS dysfunction is an early event in synucleinopathy and may represent a promising therapeutic target for the development of disease modifying treatments in PD.

Our study did not compare UPS activity to that of other cellular protein quality control pathways such as the autophagy-lysosomal pathway (ALP). The link between ALP dysfunction and PD is well-established with identification of several PD-associated genes related to the ALP (e.g. LRRK2, VPS35) and evidence from cellular and animal models that misfolded forms of α-synuclein lead to ALP dysfunction (Reviewed in [3]). Technical limitations, such as probe specificity and signal detection in brain tissue, restrict the viability of pursuing an option to directly compare and/or dissect the relative contribution of the UPS versus ALP pathways in the degradation of α-synuclein. Since UPS dysfunction was found to precede motor dysfunction and neuronal loss in the present study, it is likely to be a relevant therapeutic target, alongside the ALP, with the aim of reducing levels of misfolded proteins in dopaminergic neurons.

After the initial observation that Lewy bodies were highly ubiquitinated [27], several studies have implicated dysfunction of the UPS in PD pathogenesis. Post-mortem studies in PD patients have revealed impairment of proteasome catalytic activity [31, 33, 45] and reduced expression of proteasomal subunits [6, 31, 32] in the SNpc. Similarly, over-expression of α-synuclein has been linked to inhibition of proteasome catalytic activity in several mammalian cell lines [13, 15], human induced pluripotent stem cells [8], and transgenic α-synuclein zebrafish [37] and mouse [10] models. The present study adds to our understanding of the role of UPS dysfunction in PD by confirming that the degree of proteasome impairment caused by mutant α-synuclein in vivo is sufficient to cause a critical loss of UPS function which results in a backlog of ubiquitinated proteasome substrates. Furthermore, we demonstrate that UPS failure is unlikely to be a secondary phenomenon of advanced nigral degeneration since it precedes the onset of both behavioural dysfunction and dopaminergic neuronal loss. The latency between the onset of UPS dysfunction and neuronal loss in the present study may reflect compensation by other protein quality control systems. For example, it is possible that early UPS dysfunction induces compensatory activation of the ALP which may help to limit the accumulation of misfolded protein conformers [11, 25, 40, 47].

Early-onset dysfunction of the UPS in affected dopaminergic neurons is likely to have profound effects on cellular proteostasis. Loss of UPS activity can lead to progressive accumulation of misfolded proteins, placing an increased demand on molecular chaperones and thus compromising their role in folding newly-synthesised proteins [30]. In addition, due to its critical role in the clearance of outer mitochondrial membrane proteins, impairment of the UPS has been linked to a loss of mitochondrial quality control and greater levels of reactive oxygen species [30]. Other potential mechanisms of neurotoxicity arising from UPS dysfunction include disruption of synaptic remodelling, impairment of the ER-associated protein degradation pathway, depletion of amino acid stores and accumulation of pro-apoptotic short-lived regulatory proteins such as p53 [30].

In the specific context of PD, UPS failure could have important consequences for the cellular metabolism of α-synuclein. The protein is subject to a range of posttranslational modifications which appear to affect its aggregation propensity and degree of cytotoxicity. The principal posttranslational modification is phosphorylation at residue S129 (pS129) of α-synuclein. In normal adult rat brain, pS129 α-synuclein accounts for approximately 4% of total α-synuclein, suggesting that it is a normal by-product of α-synuclein metabolism [16]. Pulse-chase experiments have shown that pS129 α-synuclein has a much shorter half-life (~ 55 min) than non-phosphorylated α-synuclein (~ 17 h), which is a known substrate of chaperone-mediated autophagy [12, 29]. Studies in SH-SY5Y cells and rat primary cortical neurons have shown that proteasome inhibitors promote accumulation of pS129 α-synuclein, without affecting total α-synuclein levels [9, 29]. This has led to the hypothesis that pS129 α-synuclein is a short-lived substrate of the UPS. Thus, under healthy conditions, pS129 α-synuclein accounts for only a small proportion of total cellular α-synuclein due to its continual proteasomal clearance. In the context of UPS dysfunction, however, pS129 α-synuclein is stabilised and can build up to high levels in the affected cell. This hypothesis is supported by results from our study which showed selective accumulation of pS129 α-synuclein in dopaminergic neurons affected by UPS dysfunction (Fig. 7b, c). It is also possible that pS129 α-synuclein is associated with higher levels of the Ub^G76V^-GFP reporter due to a more inhibitory effect on proteasome activity. Whilst wild-type α-synuclein is known to be sufficient to inhibit isolated proteasomes [44], phosphorylated α-synuclein has been linked to greater degrees of proteasome inhibition in cultured SH-SY5Y cells [36].

Impairment of the UPS has been recognised across a range of neurodegenerative disorders including Alzheimer’s, Parkinson’s, Huntington’s and prion diseases [30]. Common to each of these disorders is the presence of oligomeric forms of misfolded proteins and a profound disruption of cellular proteostasis. A recent study by Thibaudeau and colleagues showed that amyloid-beta, α-synuclein and mutant huntingtin adopt a common 3D conformation which stabilises the closed gate conformation of the 20S proteasome, thereby blocking protein degradation [44]. A similar mechanism of proteasome impairment may have occurred following expression of A53T α-synuclein in the present study, since we observed global inhibition of substrate hydrolysis by the different 20S catalytic sites (Additional file 1: Figure S2). This raises the prospect of developing small molecules which block interaction between oligomeric forms of α-synuclein and the proteasome, to conserve UPS function.

Several small molecules have already been shown to activate the proteasome by distinct mechanisms in vitro. For example, IU1 operates by inhibiting the proteasome-associated deubiquitinase, USP14, which in turn enhances ubiquitinated substrate translocation into the catalytic 20S core particle [26]. While effective in vitro, Vaden et al. [46] did not observe changes in proteasome activity in mice administered IU1, highlighting the challenges of upregulating UPS activity in vivo*.* In contrast, rolipram is a clinically approved phosphodiesterase inhibitor which was recently shown to activate proteasome activity in a mouse model of tauopathy [34]. It functions by raising cAMP levels to activate Protein Kinase A which enhances proteasome assembly and activity through phosphorylation of proteasome subunits [34]. Further investigation into this compound and others targeting this same pathway will be necessary to determine if they have potential as therapeutics in PD.

## Supplementary information

**Additional file 1: Figure S1.** Validation of AAV-Ub^G76V^-GFP UPS reporter in SNpc of wild-type rats. On day 0, adult wild-type rats received bilateral stereotaxic injections of 2 × 10^8^ gp/mL AAV-Ub^G76V^-GFP in 2 μL into SNpc. After 3 weeks, the same animals received 0.5 μL stereotaxic injections of 4 mg/mL lactacystin or sterile water into the left or right SNpc, respectively. Animals were culled 16 h post-administration of the proteasome inhibitor. Representative images of anti-TH (cyan) and anti-GFP (green) immunofluorescent staining of coronal cryosections reveal accumulation of the Ub^G76V^-GFP reporter in SNpc dopaminergic neurons following intranigral administration of lactacystin in AAV-Ub^G76V^-GFP-treated rats. *Scale bar* 200 μm. **Figure S2.** A53T α-synuclein inhibits chymotrypsin-, caspase- and trypsin-like peptidase activities of the 26S proteasome at 1 wpi. Adult wild-type rats received stereotaxic injection of AAV-Empty into the left SNpc and AAV-A53T into the right SNpc. Animals were culled at 1 wpi and left and right midbrain tissue isolated, followed by homogenisation. (a) Chymotrypsin-, (b) caspase- and (c) trypsin-like activities were assessed by measuring fluorescence generated from cleavage of site-specific peptide substrates, adjusted to an epoxomicin-treated control. Data are percentage activity, expressed relative to mean of AAV-Empty control group (*p < 0.05; paired t-test; *n* = 6–7 per group). **Table S1.** Primary antibodies used for immunofluorescent staining. **Table S2.** Secondary antibodies used for immunofluorescent staining.

## Data Availability

The datasets used and/or analysed during the current study are available from the corresponding author on reasonable request.
