# Supplementary material for: Early-onset impairment of the ubiquitin-proteasome system in dopaminergic neurons caused by α-synuclein
Source: Acta Neuropathol Commun. 2020 Feb 14;8:17. doi: 10.1186/s40478-020-0894-0 (PMC7023783; doi:10.1186/s40478-020-0894-0)
Supplement: Supplementary file 1 — Additional file 1: Figure S1. Validation of AAV-UbG76V-GFP UPS reporter in SNpc of wild-type rats. On day 0, adult wild-type rats received bilateral stereotaxic injections of 2 × 10^8^ gp/mL AAV-UbG76V-GFP in 2 μL into SNpc. After 3 weeks, the same animals received 0.5 μL stereotaxic injections of 4 mg/mL lactacystin or sterile water into the left or right SNpc, respectively. Animals were culled 16 h post-administration of the proteasome inhibitor. Representative images of anti-TH (cyan) and anti-GFP (green) immunofluorescent staining of coronal cryosections reveal accumulation of the UbG76V-GFP reporter in SNpc dopaminergic neurons following intranigral administration of lactacystin in AAV-UbG76V-GFP-treated rats. Scale bar 200 μm. Figure S2. A53T α-synuclein inhibits chymotrypsin-, caspase- and trypsin-like peptidase activities of the 26S proteasome at 1 wpi. Adult wild-type rats received stereotaxic injection of AAV-Empty into the left SNpc and AAV-A53T into the right SNpc. Animals were culled at 1 wpi and left and right midbrain tissue isolated, followed by homogenisation. (a) Chymotrypsin-, (b) caspase- and (c) trypsin-like activities were assessed by measuring fluorescence generated from cleavage of site-specific peptide substrates, adjusted to an epoxomicin-treated control. Data are percentage activity, expressed relative to mean of AAV-Empty control group (*p < 0.05; paired t-test; n = 6–7 per group). Table S1. Primary antibodies used for immunofluorescent staining. Table S2. Secondary antibodies used for immunofluorescent staining. [file 40478_2020_894_MOESM1_ESM.docx]

Additional file 1


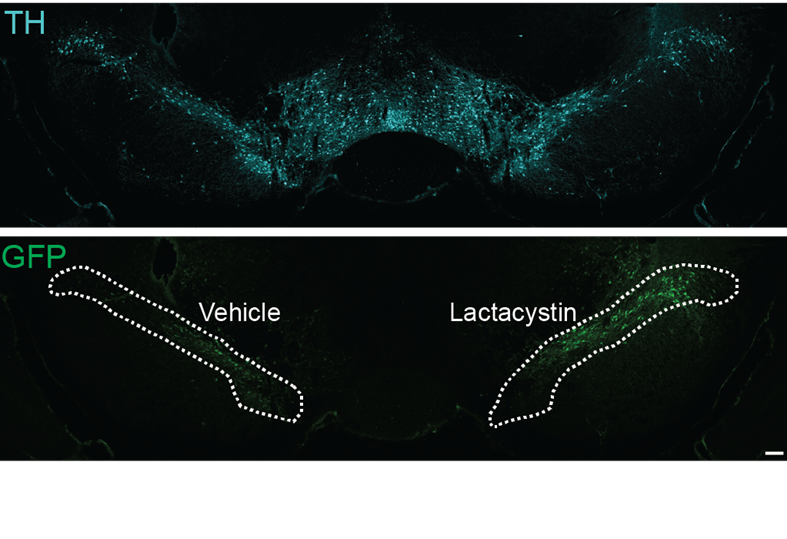


**Figure S1.** Validation of AAV-Ub^G76V^-GFP UPS reporter in SNpc of wild-type rats. On day 0, adult wild-type rats received bilateral stereotaxic injections of 2 x 10^8^ gp/mL AAV-Ub^G76V^-GFP in 2 μL into SNpc. After 3 weeks, the same animals received 0.5 μL stereotaxic injections of 4 mg/mL lactacystin or sterile water into the left or right SNpc, respectively. Animals were culled 16 hours post-administration of the proteasome inhibitor. Representative images of anti-TH (cyan) and anti-GFP (green) immunofluorescent staining of coronal cryosections reveal accumulation of the Ub^G76V^-GFP reporter in SNpc dopaminergic neurons following intranigral administration of lactacystin in AAV-Ub^G76V^-GFP-treated rats. *Scale bar* 200 μm.


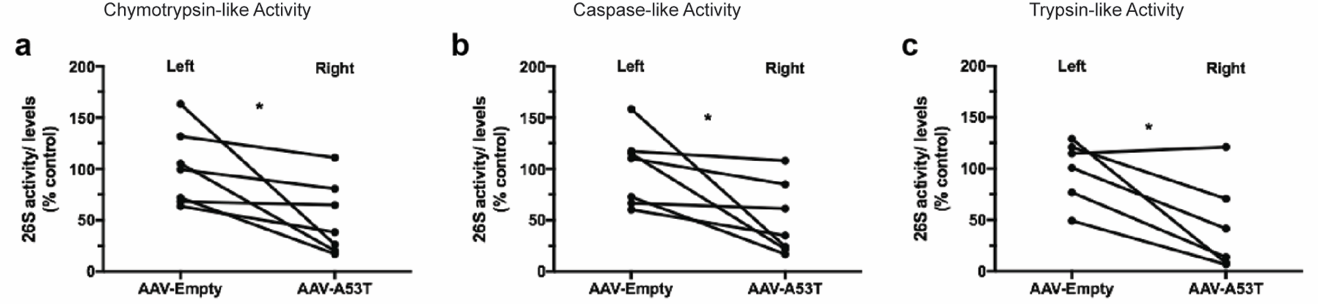


**Figure S2.** A53T α-synuclein inhibits chymotrypsin-, caspase- and trypsin-like peptidase activities of the 26S proteasome at 1 wpi. Adult wild-type rats received stereotaxic injection of AAV-Empty into the left SNpc and AAV-A53T into the right SNpc. Animals were culled at 1 wpi and left and right midbrain tissue isolated, followed by homogenisation. (a) Chymotrypsin-, (b) caspase- and (c) trypsin-like activities were assessed by measuring fluorescence generated from cleavage of site-specific peptide substrates, adjusted to an epoxomicin-treated control. Data are percentage activity, expressed relative to mean of AAV-Empty control group (*p<0.05; paired t-test; n = 6-7 per group).

**Table S1** Primary antibodies used for immunofluorescent staining

| **Antibody** | **Dilution** | **Supplier** |
| --- | --- | --- |
| Anti-GFP | 1: 200 | Invitrogen (A11122) |
| Anti-alpha synuclein | 1: 500 | ThermoFisher Scientific (32-8100) |
| Anti- alpha synuclein pS129 | 1: 1000 | Abcam (ab51253) |
| Anti-tyrosine hydroxylase | 1:1000 | Abcam (ab76442) |
| Anti-polyUb conjugates (FK1) | 1: 750 | Enzo (BML-PW8805) |

**Table S2** Secondary antibodies used for immunofluorescent staining

| **Antibody** | **Dilution** | **Supplier** |
| --- | --- | --- |
| AlexaFluor®488 Goat anti-mouse IgG | 1: 500 | Invitrogen (A11029) |
| AlexaFluor®488 Goat anti-rabbit IgG | 1: 500 | Invitrogen (A11034) |
| AlexaFluor®488 Goat anti-chicken IgY | 1:500 | Invitrogen (A11039) |
| AlexaFluor®555 Goat anti-mouse IgG | 1:500 | Invitrogen (A21424) |
| AlexaFluor®555 Goat anti-rabbit IgG | 1:500 | Invitrogen (A21429) |
| AlexaFluor®647 Goat anti-mouse IgG | 1:500 | Invitrogen (A21235) |
| AlexaFluor®647 Goat anti-chicken IgY | 1:500 | Invitrogen (A21449) |
